# Hidden Threat: Unveiling the Dangers of Cervical Epidural Abscess

**DOI:** 10.7759/cureus.69807

**Published:** 2024-09-20

**Authors:** Praneeth Ulavala, Hemalatha Bhoompally, Hari Chandana Kalangi, Sravanthi Narapaneni, Swati Baraiya

**Affiliations:** 1 General Internal Medicine, Narayana Medical College and Hospital, Nellore, IND; 2 Intensive Care Unit, Yashoda Hospitals, Secunderabad, IND; 3 General Medicine, Kamineni Academy of Medical Sciences and Research Centre, Hyderabad, IND; 4 General Medicine, Narayana Medical College and Hospital, Nellore, IND; 5 Family Medicine, Bombay Hospital and Medical Research Center, Mumbai, IND

**Keywords:** cervical spine, epidural abscess, infection, methicillin resistant staphylococcus aureus, neurologic deficit

## Abstract

Cervical epidural abscess (CEA) is an unusual but severe condition. In most cases, it can be related to infections such as the methicillin-sensitive *Staphylococcus aureus* at the sites of intravenous cannulae. The current case report describes a patient who developed a right epidural abscess at C₅-C₇, diagnosed following persistent chest pain and fever. He eventually recovered after surgical decompression with intravenous antibiotics. The diagnosis of spinal epidural abscess (SEA) is very challenging because of the nonspecific character of symptoms. Early diagnosis using leukocytosis, elevated erythrocyte sedimentation rate (ESR), and MRI is crucial for preventing irreversible neurological deficits, as this case illustrates the importance of prompt intervention and meticulous follow-up to avoid complications like irreversible neurological deficits and enable spinal stabilization.

## Introduction

Spinal epidural abscess (SEA) is a relatively rare, though severe, medical condition characterized by abscess formation in the epidural space of the spinal cord [1]. In case of late diagnosis or mismanagement, the consequences of SEA can be devastating [1]. This case report presents an overview of the cervical epidural abscess (CEA), outlining its rarity, causal factors, clinical presentation, and prognosis as a critical acute medical emergency.

The incidence of SEA is estimated to be between 0.2 and 2.8 cases per 10,000 persons yearly, with the peak age incidence in the sixth and seventh decades of life [2]. *Staphylococcus aureus *accounts for most of the causative agents, significantly in the reported cases. Predisposing factors include a wide range of immunodeficiency states, such as diabetes mellitus, AIDS, chronic renal failure, alcoholism, cancer, epidural anesthesia, spinal surgery, and trauma [3]. The treatment includes a course of intravenous antibiotics coupled with surgical intervention, which leads to the alleviation of symptoms and a successful recovery.

Although relatively uncommon, CEAs represent about 20% of all SEA cases. Their peculiar location poses diagnostic and therapeutic challenges, often giving less favorable outcomes as compared to lower epidural abscesses [4].

## Case presentation

This is a case report of a 60-year-old male patient with a history of hypertension and diabetes mellitus admitted to the hospital with complaints of chest pain and fever. It provides valuable clinical insight into the CEA. Evaluating the specifics of this case delineates the challenges that persist in diagnosing and treating SEAs and the importance of timely intervention.

Clinical presentation and diagnosis

The patient's initial presentation with chest pain, for which cardiology consultation was sought, and the fever also points out the nonspecific nature of SEA symptoms, which often may mimic other medical conditions. The tricky part in diagnosing SEA is its various clinical manifestations that can lead to missed or delayed diagnosis. In this case, admission for hyponatremia and subsequent development of thrombophlebitis on the left forearm added to the complexity of the clinical picture.

Imaging findings and neurological deficits

The development of transient right upper limb weakness during hospitalization led to the suspicion of neurological involvement. Neurological deficits are an essential feature of SEA, and once present, it should lead to a high index of suspicion for this disorder.

**Figure 1:**
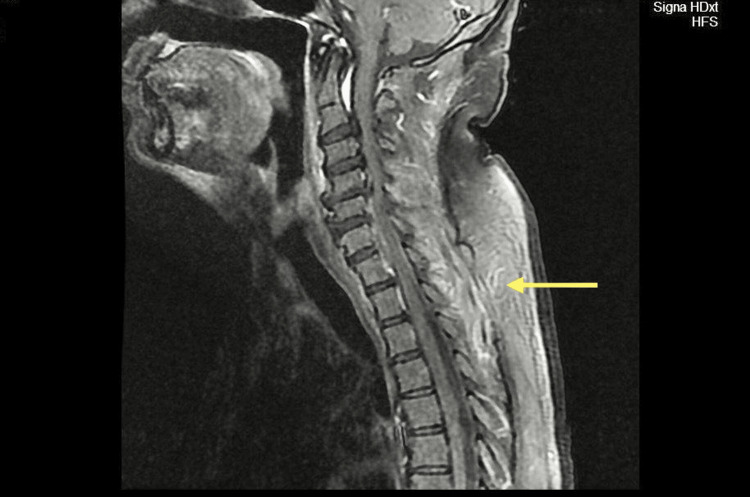
MRI of the cervical spine in sagittal view showing a CEA, as indicted by the yellow arrow. CEA: Cervical epidural abscess

**Figure 2:**
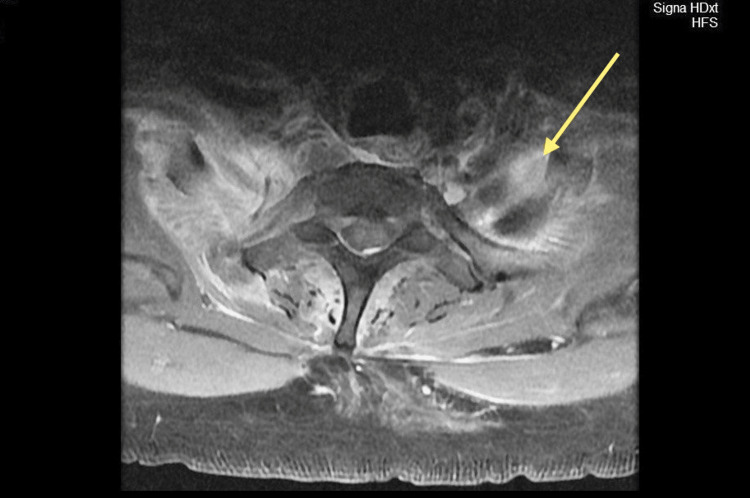
MRI of the cervical spine in transverse section showing a CEA, as indicated by the yellow arrow. CEA: Cervical epidural abscess

MRI was crucial to the diagnosis, showing a right epidural abscess at the level of C₅-C₇, with prominent bilateral cord compression as shown by the yellow arrows in Figures 1 and 2. These imaging findings represent that an MRI is a prime imaging modality, correctly representing the extent and location of the abscess for guiding surgical planning.

Causative organism and antibiotic treatment

The cultures of blood and pus isolated the causative organism as methicillin-sensitive *Staphylococcus aureus*, thus underlining the role of microbiological investigations in arriving at proper antibiotic therapy. The intravenous use of flucloxacillin 2 grams was given every six hours which was guided based on the sensitivity pattern so that the identified pathogen would be adequately covered.

Surgical intervention and outcome

Because of the significant bilateral cord compression, the patient underwent C₅, C₆ corpectomy, evacuation of the epidural abscess, and C₄-C₇ fusion with an expandable cage with anterior cervical plate and screws. Surgical decompression was done to relieve neural compression and prevent further neurological deterioration.

Gradually, with postoperative treatment on intravenous antibiotics and other supportive therapy, he improved symptomatically, with recovery of the right upper limb power. Again, this discharge of the patient in a hemodynamically stable condition signifies the importance of early intervention in avoiding severe morbidity and facilitating patient's recovery.

Prognosis and implications

This case illustrates that, if not identified and treated early, SEA can lead to very serious and irreversible neurological deficits. In contrast, timely diagnosis and early intervention, as in this report, offer the best promise for positive patient outcomes and prevention of long-term neurological disability.

## Discussion

Timely and accurate diagnosis of SEA is of paramount importance for a favorable outcome since delayed diagnosis and inappropriate management are associated with severe and sometimes irreversible neurological deficits [5]. The reported rates for correct diagnosis at the time of initial presentation range from as low as 20% to 70%. The challenge of diagnosing this condition is therefore highlighted [6]. 

Most clinical SEA manifestations are nonspecific; therefore, it is challenging to clinically differentiate it from other causes of back pain and fever. Characteristically, symptoms include backache, fever, radicular pain, limb weakness, sensory alterations, and bladder or bowel dysfunction [7]. The typical sequence of symptoms is initial backache and tenderness, progressing to root pain and sensory changes, followed by limb weakness, ascending numbness, and paralysis in severe cases [7]. 

Things can also become further complicated because not all cases are characterized by leukocytosis and an increased erythrocyte sedimentation rate (ESR), making early diagnosis difficult [8]. MRI with contrast is considered the gold standard method of imaging in SEA patients [9-11]. It provides comprehensive visualization regarding the location and extent of the abscess within the spinal canal.

With the increased number of immunocompromised patients, invasive procedures, and instrumentation, the incidence of SEA may be rising. This has also been caused by the recently evolved methicillin-resistant *Staphylococcus aureus *(MRSA), further complicating its management [2,12]. 

This case report points out that SEA emphasizes the need for awareness of clinical features and diagnostic challenges that have to be created among health care professionals. On the other hand, heterogeneous manifestations and possible misdiagnosis underline the need to consider SEA in the differential diagnosis of patients who present with back pain, fever, and neurological deficits, particularly in those patients with risk factors [10]. 

Moreover, the emergence ofMRSAas a causative agent in SEA is relevant to monitoring bacterial sensitivity patterns for the optimizing antibiotic therapy and patient care [13].

Future research efforts must be directed toward early diagnostic strategies and exploring novel treatment modalities in SEAs to improve patient outcomes and prevent irreversible neurological dam⁤age [14]. 

The purpose of this article is to review the literature on epidural abscesses with emphasis on their etiology, clinical presentation, and prognosis in this life-threatening medical emergency. Bringing attention to the predisposing factors and clinical characteristics could raise awareness that facilitates early recognition and timely neurosurgery consultation. Early diagnosis with the institution of appropriate treatment remains the cornerstone for achieving successful outcomes and avoiding devastating morbidity and mortality associated with CEAs [5].

## Conclusions

The case report gives valuable clinical insights into the diagnosis and management of CEAs. Early recognition, accurate imaging with MRI, and prompt multi-disciplinary intervention with antibiotics and surgical decompression are the keys to success. A high index of suspicion for SEA should prevail, particularly in patients with predisposing factors, to prevent severe morbidity and ensure favorable neurological recovery. Further research and vigilant clinical observation may advance our understanding of SEA and in improve the care of this rare but potentially devastating condition. 
